# EEG Alpha Power Is Modulated by Attentional Changes during Cognitive Tasks and Virtual Reality Immersion

**DOI:** 10.1155/2019/7051079

**Published:** 2019-06-25

**Authors:** Elisa Magosso, Francesca De Crescenzio, Giulia Ricci, Sergio Piastra, Mauro Ursino

**Affiliations:** ^1^Department of Electrical, Electronic and Information Engineering “Guglielmo Marconi”, University of Bologna-Campus of Cesena, Via dell'Università 50, Cesena, Italy; ^2^Department of Industrial Engineering, University of Bologna-Campus of Forlì, via Fontanelle 40, Forlì, Italy

## Abstract

Variations in alpha rhythm have a significant role in perception and attention. Recently, alpha decrease has been associated with externally directed attention, especially in the visual domain, whereas alpha increase has been related to internal processing such as mental arithmetic. However, the role of alpha oscillations and how the different components of a task (processing of external stimuli, internal manipulation/representation, and task demand) interact to affect alpha power are still unclear. Here, we investigate how alpha power is differently modulated by attentional tasks depending both on task difficulty (less/more demanding task) and direction of attention (internal/external). To this aim, we designed two experiments that differently manipulated these aspects. Experiment 1, outside Virtual Reality (VR), involved two tasks both requiring internal and external attentional components (intake of visual items for their internal manipulation) but with different internal task demands (arithmetic vs. reading). Experiment 2 took advantage of the VR (mimicking an aircraft cabin interior) to manipulate attention direction: it included a condition of VR immersion only, characterized by visual external attention, and a condition of a purely mental arithmetic task during VR immersion, requiring neglect of sensory stimuli. Results show that: (1) In line with previous studies, visual external attention caused a significant alpha decrease, especially in parieto-occipital regions; (2) Alpha decrease was significantly larger during the more demanding arithmetic task, when the task was driven by external visual stimuli; (3) Alpha dramatically increased during the purely mental task in VR immersion, whereby the external stimuli had no relation with the task. Our results suggest that alpha power is crucial to isolate a subject from the environment, and move attention from external to internal cues. Moreover, they emphasize that the emerging use of VR associated with EEG may have important implications to study brain rhythms and support the design of artificial systems.

## 1. Introduction

The preeminent oscillatory phenomenon in brain neurodynamics is represented by the alpha rhythm (approximately 8–12 Hz), which is the dominant frequency in the human scalp EEG [1]. It is well known that EEG activity in the alpha band exhibits a significant change in a variety of conditions; depending on the kind of stimulus or task demand, a brain region responds either with a decrease in alpha power (Event-Related Desynchronization, ERD) or an alpha power increase (Event-Related Synchronization, ERS) [2, 3]. More particularly, a large body of the literature suggests that regions activated during a task exhibit ERD, whereas ERS occurs in regions irrelevant for the task, or regions which process distractors or potentially interfering cues [4–7].

Furthermore, recent studies propose an interpretation of human alpha rhythm in terms of a distinction between internally and externally directed attention.

For what concerns external attention, it is well known that alpha power decreases over occipital sites during visual stimulation [8] and over sensorimotor areas during sensorimotor tasks or movements [3]. Various studies relate the level of visual attention to the strength of oscillatory *α* activity, observing that greater external attention causes a decrease in alpha power, or a shift in the alpha rhythm toward the attended locations [9–11]. Alpha desynchronization has been associated to tasks requiring processing of relevant information in a variety of cognitive domains, but especially associated with visual perception [12–14]. It was thus hypothesized that the suppression of alpha activity is related to the strength of attention to external objects or stimuli required by the task [15].

Different results, however, have been recently observed in the auditory domain, where an increase of alpha has been linked to increased effort and/or processing [16–19]. Hence, the role of a task on the alpha-band power, even during processing of external inputs is still strongly debated, with possible significant differences in auditory and visual domains and in different tasks.

Conversely, *a*-band oscillations have been observed to increase during internal tasks, such as visual imagery or arithmetic operations [20–23]. An old influential hypothesis by Ray and Cole [24] assumes that alpha power increases during rejection tasks (internally directed attention), to reflect inhibition or rejection of incoming sensory information. Since an inward shift of attention is accompanied by an increase in alpha power, some authors suggested that ERS may be working to inhibit sensory processing and suppress distractors or potentially interfering cues [4–7, 25, 26] or more generally to implement a general inhibitory mechanism in the brain [26, 27].

Consequent to the previous observations is the idea that *α*-power modulation is strictly related with working memory (WM). During WM tests, selective attention may be operating to enhance the efficient use of limited memory resources, by enabling the encoding of relevant information and avoiding that memory capacity is degraded by interfering cues [28]. Indeed, several studies have shown a significant *α*-synchronization associated with memory load in experiments in which participants were presented items to be remembered for a short period [25, 29, 30]. An influential hypothesis is that alpha oscillations work as a filter mechanism able to inhibit an increasing number of distractors via a progressive *α*-power increase [31]. However, divergent results have been reported on this point. While Sauseng et al. [32] found that alpha activity increases with the number of distractors, other failed to report these changes [33, 34].

From the previous literature, we can conclude that while the relationship between alpha activity and attention mechanisms is well documented today, both when attention is directed toward external stimuli (ERD) and remembered items (ERS), the exact role played by alpha oscillations and its modulation by the task is still unclear. In particular, the alpha-inhibition hypothesis and the role of alpha activity during internal memory tasks continue to be questioned (for a recent review, see [35]). At least three important elements are involved in these procedures: maintenance of internal memory, processing of external stimuli, and task load requirements. As pointed out by van Moorselaar et al. [36], it is still unclear whether these aspects cooperate or are in conflict, and how they interact at the frontal and occipital level to ensure the better behavioural performance. Does alpha activity reflect an internal cognitive process, under the influence of top-down mechanisms which work to focus attention on the essential items, i.e., a shift between “bottom-up” and “top-down” requirements, as suggested by von Stein et al. [37, 38]? Or does it simply reflect the disengagement of attention from external stimuli? Does alpha desynchronization signal a major role of external sensory representation, whereas alpha synchronization emphasizes a major role of internal mental processing?

In order to examine these aspects, we need experiments which manipulate both external stimulation, cognitive processing requirements (i.e., task difficulty), and direction of attention (external vs. internal). In particular, we wish to investigate in which terms alpha power can be reduced by tasks which require an attentional focus to external items, how this desynchronization is affected by the task demand, and how it is affected by strong external stimulation in absence of specific tasks and finally by a mental process which requires isolation from the environment.

To reach our objective, the study comprises two subsequent but strictly related experiments: (i) changes in alpha-band power (i.e., ERD or ERS) were measured in laboratory, using a 13-electrode system, during two tasks which differently recruited visual and cognitive mechanisms (the first is a reading numbers task, the second a visual + arithmetic operation task). The results are used to assess ERD during attentive tasks that require external attention, and its modulation by the level of attention/involvement required. (ii) Changes in alpha-band power were quantified when participants interact with a business aircraft cabin in a Virtual Reality (VR) setting, to mimic conditions experienced by a passenger during an airplane travel. In this case, the EEG was obtained while the participant was immersed in a VR environment, conceived to simulate the main visual and acoustic characteristics of a cabin interior, ad hoc designed during the project. We assume that this condition strongly solicits the external visual/acoustic attention, even in the absence of a specific task. Finally, in the same condition (VR immersion), we asked the participants to perform a mental arithmetic task (internal attention) and to investigate the conflict between the external virtual immersion and the internal focus and its effect on alpha rhythm power.

In all cases, alpha rhythm was investigated both at the parieto-occipital and frontal regions, to point out differences.

Finally, we wish to stress that a novel aspect of this work is the analysis of alpha rhythm in VR environment. The study was designed within the framework of the Horizon 2020 project CASTLE (CAbin Systems design Toward passenger welLbEing), aimed at optimizing the design of innovative interiors of aircraft cabins for Business Jet Industry, also exploiting VR for the collection of users' feedback. Indeed, the present availability of sophisticate VR instruments now allows changes in brain rhythms to be studied when the subject is immersed in a complex realistic scenario and mimicked in a controlled repeatable condition. This idea opens new perspectives not only in the design of artificial systems, but also in the study of the human interaction with the external world.

## 2. Materials and Methods

Two experiments were carried out in the present study. They served to differently manipulate the task load (more/less demanding task) and direction of attention (internally/externally directed attention). The first experiment (Experiment 1) was performed in a controlled laboratory environment outside the VR setting; a classical monitor screen was used for stimuli presentation to participants and a wired EEG device was used for signal collection. Experiment 1 included two tasks that both required external and internal attentional components (intake of visual items for their internal manipulation) but with different task loads, one task being more demanding than the other. In the second experiment (Experiment 2), we took advantage of the VR technology to strongly manipulate the direction of attention. This experiment was conducted in a VR laboratory where participants were exposed to and interacted with a VR environment (aircraft business cabin interior), and a wireless EEG device was used for data collection. Experiment 2 involved a condition consisting in purely VR immersion whereby the rich sensory stimulation elicited external attention and a condition consisting in performing a mental task during the VR immersion; at variance with purely VR immersion, the latter condition required internal attention and neglect of external environment to perform the mental operations.

### 2.1. Participants

Thirty healthy volunteers (10 females), aged 20–42 years (mean ± std = 25.4 ± 4.8 years), took part in Experiment 1. Forty-one healthy volunteers (9 females), aged 19–29 years (mean ± std = 22.1 ± 2.6 years), took part in Experiment 2. Participants in the two experiments were different; this avoided that the participants were subjected to a long recording involving several sessions and conditions (of both experiments), that may have induced tiredness and boredom. Each participant had normal or corrected to normal vision and reported no medical or psychiatric illness. The study was approved by the local ethical committee (file number: 187339, year: 2018), and all participants gave written informed consent before the beginning of the experiment. All data were analyzed and reported anonymously.

### 2.2. Experiment 1: Cognitive Tasks Driven by External Stimuli and with Different Demand

#### 2.2.1. Experimental Protocol

**Figure 1:**
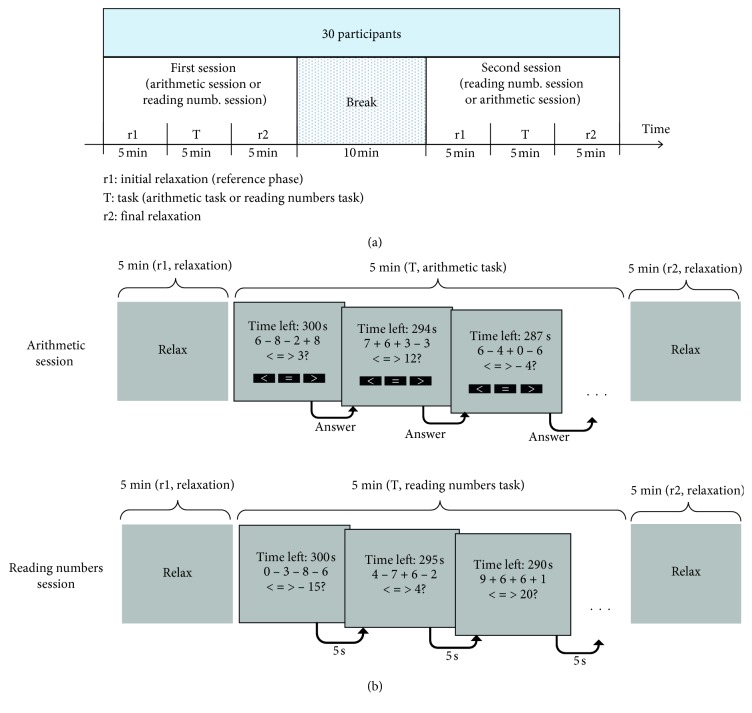
(a) Timeline of Experiment 1. The experiment included two sessions, i.e., an arithmetic session and a reading numbers session, separated by a 10 min break, performed by all participants. Each session lasted fifteen minutes and included an initial (r1) 5-minute relaxation phase, a final (r2) 5-minute relaxation phase, and a central 5-minute task phase (T) consisting in an arithmetic task (arithmetic session) or a reading numbers task (reading numbers session). The order of the tasks was counterbalanced across the participants. (b) Design of each session. In both sessions, the relaxation phases (r1 and r2) consisted in the presentation of a gray screen with the world “relax.” In the arithmetic session, during the task phase, the participant had to provide the response to the arithmetic operation (by selecting one of the black button-items with the mouse); after the response selection, a new screen with a new operation appeared. In the reading numbers session, during the task phase, the participant had just to mentally read the numbers appearing on the screen (e.g., 300, 0, 3, 8,…), and the screen updated every 5 seconds.

The participants comfortably seated facing a computer monitor at about 50 cm far, in a dedicated laboratory. They underwent two experimental sessions, each lasting 15 minutes, separated by a break of about 10 minutes (Figure 1(a)). Each experimental session consisted of three phases: a 5 min *initial relaxation* phase (named r1), a 5 min *central task* phase (named T), and a 5 min *final relaxation* phase (named r2). The two relaxation phases, preceding (r1) and following (r2) each task, were identical in both sessions: a gray screen with the word “relax” was continuously displayed (Figure 1(b)), and participants were instructed to relax during such phases maintaining the eyes open. The experimental sessions differed only in the type of the task executed during the central phase, namely, an *arithmetic task* and a *reading numbers task* (Figure 1). The order of the tasks was counterbalanced across participants. Both the implemented tasks involved exploration and intake of visual items (symbols and numbers) and their internal manipulation; thus, they involved both visual-spatial processes (external attentional component) and cognitive processes (internal attentional component), but the arithmetic task required higher level of sensory attention and cognitive effort.

*(1) The Arithmetic Task*. During this task, the participants had to solve the arithmetic operations displayed on the screen, consisting in the addition/subtraction of four one-digit numbers, and had to compare the result with a given displayed target. They provided their response by selecting one of the three displayed button-items (black boxes with symbols < = >, see Figure 1(b)) using the mouse. Each operation was displayed on the computer monitor continuously until the participant responded; immediately after, the screen was updated displaying a new operation together with the target and the three response items (Figure 1(b)). Participants were instructed to answer not only as accurately as possible but also as quickly as possible, motivated by a timer that signaled the time left at each screen update (Figure 1(b)). For each arithmetic operation, the four one-digit numbers and the three operators (+ or −) were generated randomly; the comparison target was generated as a random integer close to the correct result of the arithmetic operation in order to avoid trivial solutions (the absolute difference between the comparison target and the correct result was ≤3).

*(2) The Reading Numbers Task*. During this task, the screen displayed the arithmetic operation, the comparison target, and the timer in order to provide similar visual items as in the arithmetic task, but the participants were clearly instructed to just mentally read the numbers presented on the screen, without performing any operation (response buttons were not displayed). The screen was updated every 5 seconds (Figure 1(b)). At each screen update, the numbers and operators in the arithmetic operation and the comparison target were generated randomly as in the arithmetic task.

Tasks similar to the ones implemented here were previously adopted in other studies to investigate attentional-related EEG rhythms modifications [39–41].

Before the onset of each experimental session, the participants received the instructions about the task of that session. During each session, participants were asked to reduce body and head movements at minimum (except finger movement for mouse use in the arithmetic task) and not to speak.

It is worth noticing that, in each session, the relaxation phase r1 was considered as the reference state within that session, and the alpha power modifications induced by the task in the following phases T and r2 were evaluated with respect to this reference state (see also Section 2.2.3). This was done to focus only on the changes induced by the specific task, ruling out other possible confounding effects (e.g., participant's fatigue due to execution of the previous session).

#### 2.2.2. EEG Recording and Preprocessing

During each experimental session, thirteen EEG signals were recorded via a wired, laboratory-grade device (Brainbox® EEG-1166 amplifier, Braintronics, The Netherlands and Neurowave Acquisition Software, Khymeia, Italy), using wet Ag/AgCl scalp electrodes (embedded in an elastic cap). Electrodes were located at positions F3, F4, T7, C3, Cz, C4, T8, PO7, PO3, PO8, PO4, O1, and O2; the reference electrode was placed on the right earlobe, and the ground electrode was located on the forehead. The number and positions of the electrodes were chosen as a trade-off between the following requirements. (i) Use of a restricted number of electrodes in an effort to outline a system characterized by ease of use, reduced setup time, and low cost, prospectively aimed at real-world practical applications. (ii) Allow coverage of both the frontocentral and parieto-occipital regions, the latter known to be more involved in visual-spatial (and computational) processing than the first [21, 39, 42, 43]. This may be useful to detect potential differences among scalp regions.

During each experimental session, the EEG signals were digitized at a sample frequency of 128 Hz and 16 bit resolution, and with the inclusion of a hardware notch filter eliminating line noise at 50 Hz. Then, for each participant, the two 15-minute EEG recordings, each relative to one of the two different sessions, were converted in a Matlab-compatible format for further offline processing (Matlab R2016a, The MathWorks Inc., Natick MA). First, each 15-minute recording was high-pass filtered at 0.75 Hz to eliminate the DC offset and slow drifts. Subsequently, we applied the Independent Component Analysis (ICA), an effective method largely employed for removal of artefacts from EEG [44–46]. For this purpose, each recording was entered into the “infomax” ICA algorithm (implemented by the EEGLAB toolbox) [47, 48]; artefactual Independent Components were visually identified and removed. An average of 3.87 ± 0.8 Independent Components were rejected across all participants and sessions. In particular, three rejected components were common across all recordings and separated three independent artefact activities inevitably present, i.e., eye blink, lateral eye movements, and heartbeat; one or two additional artefact components were occasionally present extracting EMG-related activity or single-channel noise.

#### 2.2.3. Alpha Power Computation

For each participant and each session, the preprocessed EEG signals were subdivided into three parts of 5 minutes each, corresponding to the three phases of the session (r1, T, and r2). The Power Spectrum Density (PSD) of each channel over each phase was obtained by applying Welch's periodogram method, by using a Hamming window of 5 seconds at 50% overlap, zeropadded to 10 s to obtain 0.1 Hz frequency resolution. Then, for each channel, the power in the alpha band 8–12 Hz was computed for each phase r1, T, and r2. Moreover, a normalization procedure was adopted. Specifically, in each session, the alpha power value of a single channel in the r1 phase was used as reference value for that channel, and the alpha power in each phase of the same session was divided by this reference value, to obtain the normalized alpha powers for that channel.

In addition to the analysis at single-channel level, we performed an analysis at scalp-region level, by aggregating the channels into two regions of interest: a region (fronto-central-temporal, *FCT region*) including the anterocentral channels (F3, F4, T7, C3, Cz, C4, and T8) and a region (Parieto-occipital, *PO region*) including the posterior channels (PO7, PO3, PO8, PO4, O1, and O2). To this aim, for each participant and each session, the mean PSD over the FCT and PO regions were computed by averaging the PSD across the corresponding channels, separately for each phase r1, T, and r2. Then, similarly to the single-channel analysis, the power in the alpha band 8–12 Hz was computed over each region and for each phase r1, T, and r2. Finally, the normalized alpha powers at the scalp-region level were computed: the alpha power in the r1 phase over a region was used as the reference value for that region, and the alpha power value in each phase over the same region was divided by this reference value. Of course, the normalized alpha powers assumed value 1 in the r1 phase, both at single-channel level and at scalp-region level.

### 2.3. Experiment 2: VR Immersion and Mental Task in VR Immersion

#### 2.3.1. Virtual Reality Instrumentation and the Aircraft Virtual Cabin Interiors

**Figure 2:**
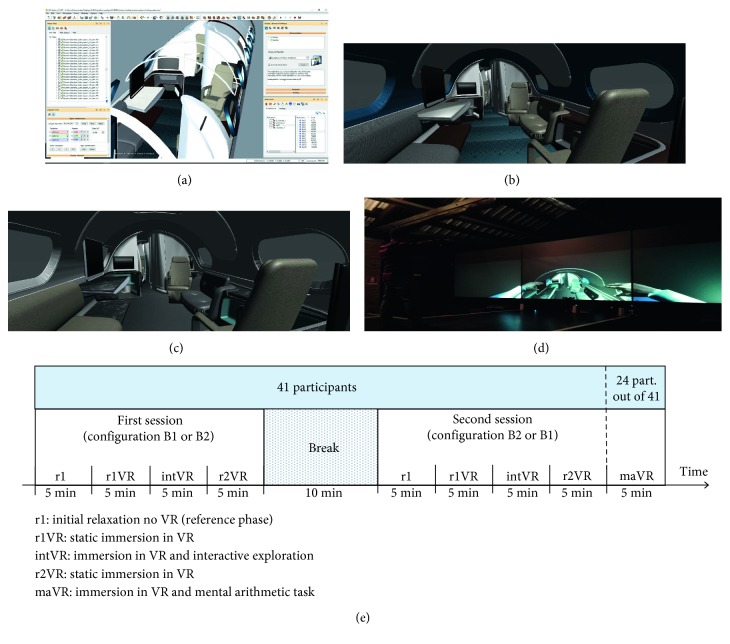
(a) CAD model processed in IC.IDO (Industrial Grade Immersive VR Solutions) Software creating the digital mock-up of the entire cabin with the proper color, material, and finishing properties of each surface. (b, c) The two different configurations of the cabin interior, namely, configuration B1 (b) and B2 (c), characterized by different color, material, and finishing properties, when projected on the CAVE screens. (d) Example of the avatar within the cabin virtual environment. (e) Timeline of Experiment 2. The experiment included two sessions corresponding to the cabin configurations B1 and B2. The order of the presentation of the two configurations was counterbalanced across participants. All participants executed the phases r1 (relaxation without VR), r1VR (first static immersion in the VR), intVR (interactive exploration of VR), and r2VR (second static immersion in the VR) in both sessions. Only a subset of participants (24 out of 41) executed an additional phase in the second session (phase maVR), consisting in performing a mental arithmetic task (mental serial subtractions) while immersed in the VR.

The concept and the CAD (Computer Aided) model of the cabin interiors of a business jet were provided by ACUMEN (https://acumen-da.com/). The model design is based on a modular layout of the cabin that is divided into five zones, and for each zone, different functional requirements have been defined by Dassault Aviation. There is a flexible area for informal and formal activities. Moreover, there is a rear cabin area designed with enough privacy and discretion as main targets. The central lavatory is between the two flexible zones and is expected to be easy to access to and safe to use. Finally, the galley and the crew rest areas are provided, all referenced in the fuselage model. The surface CAD model was processed in IC.IDO (Industrial Grade Immersive VR Solutions) Software to create the digital mock up of the entire cabin with the proper color, material and finishing properties for each visible surface. IC.IDO® is a 3D immersive VR software, provided by ESI® Group, supporting industrial decision making processes and digital mock-up verifications (Figure 2(a)). Then, two different CMF (color, material, and finishing) configurations of this cabin model were prepared for test (Figures 2(b)–2(c)), namely, configurations B1 and B2.

The cabin model files, properly converted and refined, were deployed on the CAVE (Cave Automatic Virtual Environment) at the Virtual Reality Laboratory of the University of Bologna. The CAVE is a multiple screens stereoscopic visualization system that immerses the user in a virtual environment [49]. The CAVE is developed on top of Commercial of The Shelf (COTS) components and is based on three 2.5 × 1.9 m rear-projected screens and a floor. The active stereoscopy was enabled through shutter glasses. To allow the cabin environment to be navigated from a first-person perspective by a user moving on the CAVE floor, face and body tracking was implemented by capturing and filtering data provided by a Microsoft Kinect sensor placed in front of the user at the bottom of the CAVE central screen. Tracking of the face was used to update the VR camera's point of view with the actual user's point of view [50]. Body tracking allows the longitudinal navigation of the cabin, implemented through the amplification of the user's step distance in the main axis direction. In addition, an avatar representing the user was introduced in the cabin virtual environment, and the avatar's joints and face position and orientation were linked to the user's ones captured by Kinect, so that the avatar replicated user's movements and gestures (Figure 2(d)). Finally, to simulate interaction with objects of the virtual environment, a sound was produced by the system whenever the avatar hurt or touched them, to fake collision.

#### 2.3.2. Experimental Protocol

The participants underwent two experimental sessions within the VR laboratory, one for each virtual cabin configuration, B1 and B2, separated by a break of about 10 minutes (Figure 2(e)). The order of the presentation of the two configurations was counterbalanced across participants. It is worth noticing that the replication of the session using two virtual configurations of the same environment served to test the robustness of the adopted EEG measure (alpha power) and of its extraction procedure. Indeed, as the two configurations differed in subtle details (color and finishing), we expected similar sensory stimulation to be elicited by immersion in them and therefore similar effects on alpha power to be observed across the sessions (see also Sections 2.3.4 and 3.2.1).

Throughout each session, lights were kept off to improve clearness and contrast of the images projected on the CAVE screens and favor participants' immersion within the VR environment; moreover, a background airplane sound was played continuously. All participants were required to not speak throughout the sessions.

Each session was structured into 5-minute phases. The two sessions shared the same structure with the exception that 24 out of the 41 participants performed an additional phase (maVR) in the second session (with either the B1 or B2 virtual configuration, see Figure 2(e)). This additional phase served to test the effects of an internal task (mental arithmetic) requiring isolation from the realistic external context. The remaining phases were common across the two sessions for all participants (Figure 2(e)). The first 5-minute phase, named r1, consisted in an *initial VR-off relaxation phase* without VR stimulation (and only the background sound on); during this phase, the participants were seated centrally in front of the black screens at a distance of about 2 meters and were instructed to relax with eyes open, while the VR environment was kept off. Immediately at the end of this phase, the VR environment was turned on and was kept on until the end of the session. The second 5-minute phase, named r1VR, consisted in a *first still (static) VR immersion*: during this phase, the participants remained seated while being immersed in a static VR scenario, showing the cabin lounge and conference room (flexible area), and were solicited by the rich sensory stimuli and free to visually explore the virtual scenario (via eye and head movements). The third 5-minute phase, named intVR, consisted in *an interactive VR exploration*: during this phase, the participant stood up, walked, moved, and interacted through the virtual cabin interior, trying to explore all the zones. The fourth 5-minute phase, named r2VR, consisted in a *second still (static) VR immersion,* following the interaction phase: during this phase, the same conditions as in the r1VR phase were replicated, with the participants seating again immersed in the same static scenario shown previously. The additional phase maVR performed by the subset of participants consisted in a mental arithmetic task executed during the VR immersion: during this phase, the participant remained seated immersed in the same scenario as in r1VR and r2VR and performed mental serial subtractions in steps of seventeen starting from 1000.

In this study, we did not employ a realistic seat (i.e., similar to the ones present in a real cabin) during the phases in which participants remained seated; of course, this improvement could be implemented in future studies to further enhance the VR experience.

The relaxation phase r1 in each experimental session was considered as the reference condition within that session, and the modifications induced by the VR immersion as well as by the mental arithmetic task (phases r1VR, r2VR, and maVR) were evaluated with respect to this reference state (see also Section 2.3.4), to exclude possible bias due to execution of the previous session. It is worth noticing that, for each session, the interactive exploration phase, intVR, was excluded from the analysis (see also Section 2.3.3). Indeed, this phase mainly included motor aspects which fall outside the focus of the present study (moreover, this analysis would be particularly complex as removing locomotion-induced mechanical artifacts from EEG signals in a reliable way is still a critical problem). Rather, the interaction phase might be useful to assess whether alpha power was modified before and after an active exploration of the VR environment, possibly reflecting a modification of external attention level.

#### 2.3.3. EEG Recording and Preprocessing

In this experiment, a wireless consumer-grade EEG device was used to acquire the EEG signals. Specifically, we employed the OpenBCI Cyton board complemented with the OpenBCI Daisy Module (OpenBCI, https://openbci.com/) that allows up to 16 differential EEG channels to be acquired wirelessly via the OpenBCI USB transmitter/receiver using RFduino radio module. The use of a wireless device was fundamental for EEG recording in the VR laboratory, eliminating restrictions on positioning the participants inside the laboratory and allowing free movements and mobility of the participant when immersed in the VR scenario.

Twelve wet Ag/AgCl electrodes (F3, F4, T7, C3, C4, T8, PO7, PO3, PO8, PO4, O1, and O2) of an electrode cap were plugged into the differential channels of the OpenBCI Cyton + Daisy Board, and the board was secured over the cap in the central position, so as to realize a wireless and wearable system. The same electrodes as in Experiment 1 were used, except electrode Cz skipped for board fixing. The reference electrode was placed on the right earlobe and the ground (bias) electrode was placed on the left earlobe.

For each participant and during each experimental session, the twelve EEG signals were online digitized at a sample frequency of 125 Hz and 24 bit resolution and stored in a Matlab-compatible format. Then, each recording was offline preprocessed. First, each recording was high-pass filtered at 0.75 Hz to eliminate the DC offset and slow frequency drifts and filtered by a 50 Hz notch filter to eliminate line-power interferences. Then, the portion of the signals corresponding to the interaction phase (intVR, from minute 10 to minute 15) was excluded from any further analysis, and the signals in the remaining phases (r1, r1VR, r2VR, and the additional phase maVR for the subset of participants) were examined for artefacts reduction. At variance with Experiment 1, ICA applied to signals acquired in Experiment 2 was in general unable to separate artefactual activities. The reason was due to the different recording modality and device (wireless vs. wired and consumer-grade vs. laboratory grade) and different experimental conditions (participants free to move head, neck, and possibly even trunk to explore the wide facing screens vs. participants facing 15 inches monitor and instructed to reduce their movements to a minimum). As a consequence, several nonstereotypic types of noise, such as complex movement artifacts, electrode pops, transient reduction, and loss of signal transmission, affected signals in Experiment 2, besides more stereotypical artefacts (such as blinking or heartbeat related artefacts). Since only twelve ICs were returned as output, the manifold single-artefactual activities were spread over several (or even all) components, mixing with the useful signal components. Therefore, to reduce artefact effects (especially those induced by less stationary activities), we opted for a direct visual inspection of each EEG recording, removing those fragments containing muscle activities, movement artefacts, electrode artefacts, and transient lost/decreased transmission (removal was obtained by just concatenating the preserved portions). The average number of removed fragments was 2.12 ± 3.1 with a mean duration of 32 s, across participants and sessions. While the ineffectiveness of ICA may be considered a limit, this also hints practical implications. Indeed, this suggests that other procedures for artefact removal are more apt to be used in a low-density, wireless, and wearable system (and in real-world applications) and more susceptible to an online implementation, rather than ICA that requires training using sufficiently long and stationary signals.

#### 2.3.4. Alpha Power Computation

We implemented alpha power computation for the entire set of participants (41) over the two sessions and an additional computation over the second session for the subset of participants (24) who performed the additional maVR phase.

*(1) Alpha Power Computation over the Entire Set of Participants (41) and the Two Sessions (Phases r1, r1VR, and r2VR)*. This analysis served to assess the effect of purely VR immersion on alpha power. For each participant and each session, the preprocessed EEG signals were subdivided into three parts, corresponding to the three phases r1, r1VR, and r2VR (each part lasted approximately 5 minutes depending on the removed fragments), which were the phases performed by all participants in both sessions. Hence, the PSD of each channel over each phase was obtained by applying Welch's periodogram method, adopting the same parameters as in Experiment 1. The power in the alpha band 8–12 Hz was computed both at single-channel level and scalp-region level, according to the same procedure as in Experiment 1. Here, the fronto-central-temporal region was obtained by aggregating six (F3, F4, T7, C3, C4, and T8) rather than seven electrodes, as the Cz electrode was not used (see Section 2.3.3). As in Experiment 1, for each participant and for each experimental session, the alpha power values of each single channel/region in the three phases (r1, r1VR, and r2VR) were divided by the corresponding reference value (i.e., the alpha power in the r1 phase), to obtain the normalized alpha powers and to assess the alpha power modifications with respect to the reference state (r1).

Moreover, across all 41 participants, we included a further analysis to evaluate alpha power modifications at a finer time resolution. To this aim, for each participant and session, the first 10 minutes of the session (comprising the consecutive phases r1 and r1VR) were subdivided into 1-minute segments, and the alpha power over the FCT and PO scalp regions was computed with 1-minute time resolution. In this analysis, we still adopted a normalization by division using the alpha power value obtained in the first minute of the session (i.e., the first minute of the r1 phase) as the reference value.

It is important to note that the computations above were performed separately over each session obtaining separated values of normalized alpha powers for the B1 configuration and B2 configuration. In a preliminary analysis, we did not found significant differences in the B1 vs. B2 normalized alpha powers at any phase and region, in line with our expectations based on the limited dissimilarities between the two configurations. Therefore, the normalized alpha powers for the B1 and B2 configurations were collapsed together; to this aim, we computed the average alpha power across the two configurations for each participant. The collapsed values are shown in Results and used for subsequent statistical analyses (see Section 2.4).

*(2) Alpha Power Computation over the Subset of Participants (24) in the Second Session (Phases r1, r1VR, r2VR, and maVR)*. For these participants, we added a further analysis limited to the second session that included the phase maVR. This analysis served to assess how alpha power was modulated when a mental process required inward shift of attention and isolation from the environment. For each participant, the power in the alpha band 8–12 Hz was computed at scalp-region level in the four phases r1, r1VR, r2VR, and maVR of the second session. Then, for each participant and region, the alpha powers in these phases were divided by the corresponding reference value (i.e., the alpha power in phase r1), to obtain the normalized alpha powers.

### 2.4. Statistical Analyses

In both Experiments, the variable under statistical tests was the normalized alpha power obtained at the scalp-region level. For each experiment, the differences between the reference value (1) and the other phases (or times, in case of the analysis at 1 min time resolution) were tested via multiple one-sample *t*-tests, separately within each region, using Bonferroni correction (significance threshold = 0.05/*n*, where *n* was the number of comparisons). Moreover, the normalized alpha power was analyzed via repeated measures two-way Analysis of Variance (ANOVA). In Experiment 1, we analyzed the variable at the phase T and the within subject factors were: Task Type (arithmetic/reading numbers) and Region (FCT/PO). In Experiment 2, the within subject factors were: Phase (r1VR/r2VR for the variable computed on the entire set of participants; r1VR/r2VR/maVR for the variable computed on the subset of participants) and Region (FCT/PO). Post hoc comparisons were performed via pairwise *t*-tests with Bonferroni correction (significance threshold = 0.05/*n*, where *n* was the number of comparisons). For clarity, in one-sample and paired *t*-tests uncorrected *p* values were reported, together with the adjusted significance threshold.

## 3. Results

### 3.1. Experiment 1: Effect of Cognitive Tasks Driven by External Stimuli and Different Task Demands

**Figure 3:**
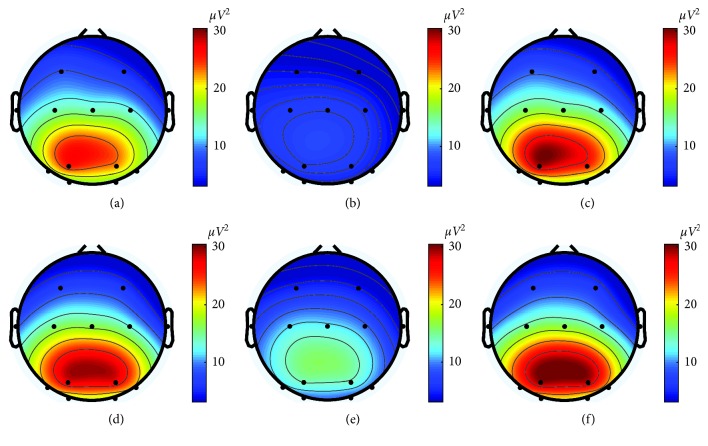
Scalp maps of alpha power (*μV*^2^) averaged across all participants in Experiment 1, as a function of the experimental session (arithmetic session: first row; reading numbers session: second row) and of the phase within the session (relaxation pretask r1: first column; task T: second column; relaxation posttask r2: third column). Each scalp map was obtained via the EEGLAB Matlab Toolbox, by color coding the average alpha power value at each electrode position in a 2D circular view (top view of the head, nose at the top) and using interpolation on a fine 67 × 67 grid. (a) Arithmetic session (r1 phase). (b) Arithmetic session (T phase). (c) Arithmetic session (r2 phase). (d) Reading numbers session (r1 phase). (e) Reading numbers session (T phase). (f) Reading numbers session (r2 phase).

Figure 3 shows the topographical scalp maps of the alpha power (not normalized) averaged across participants as a function of the experimental session (arithmetic and reading numbers session) and of the phase (r1, T, r2) within the session. In both sessions, the pretask relaxation phase (r1) was characterized by a large predominance of alpha power over the posterior area and a gradual decline towards the frontal-central regions (Figures 3(a) and 3(d)). During the task phase (T), the alpha power exhibited a widespread reduction, larger over the posterior area than over the frontocentral area; moreover, the arithmetic task (Figure 3(b)) induced a stronger alpha power decrease than the reading numbers task (Figure 3(e)). Finally, during the posttask relaxation phase (r2), the alpha power distribution resumed a similar pattern as in the r1 phase, with the alpha power increasing up to values slightly above the pretask phase (see Figures 3(c) and 3(f)).

**Figure 4:**
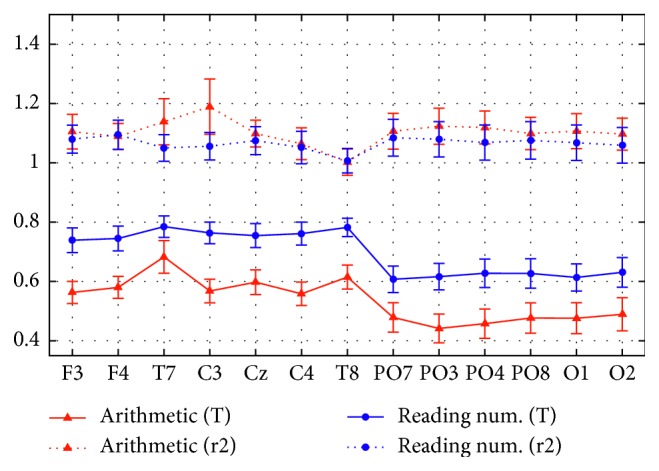
Normalized alpha power, averaged across participants (mean ± sem), at each single electrode in Experiment 1, distinguishing between session (arithmetic session: red lines; reading numbers session: blue lines) and phase (phase T: continuous lines; phase r2: dotted lines). Value 1 represents the pretask reference value (at phase r1) for each electrode; thus, values below 1 indicate alpha power decrease (desynchronization) while values above 1 indicate alpha power increase (synchronization) compared to the pretask phase, at single-channel level.

A straightforward quantification of the task-induced alpha power changes across the electrodes was obtained via the normalized alpha power at the single-channel level.  Figure 4 displays the normalized alpha power at each electrode, averaged across participants (mean ± sem), plotted during the task (T), and after the task (r2) in the arithmetic and reading numbers sessions. Thus, in this plot value 1 represents the pretask reference value for each electrode. The alpha power exhibited a larger decrease (by about 0.15 points) during the arithmetic task than that during the reading numbers task across all electrodes (solid red and blue lines). Furthermore, in the task phase (both for arithmetic and reading numbers), an abrupt reduction in the normalized alpha power was evident at the transition from the fronto-central-temporal electrodes to the parieto-occipital electrodes. During the posttask relaxation phase (r2 and dotted red and blue lines), the normalized alpha power assumed similar values across the electrodes and sessions, slightly overcoming the pretask value.

**Figure 5:**
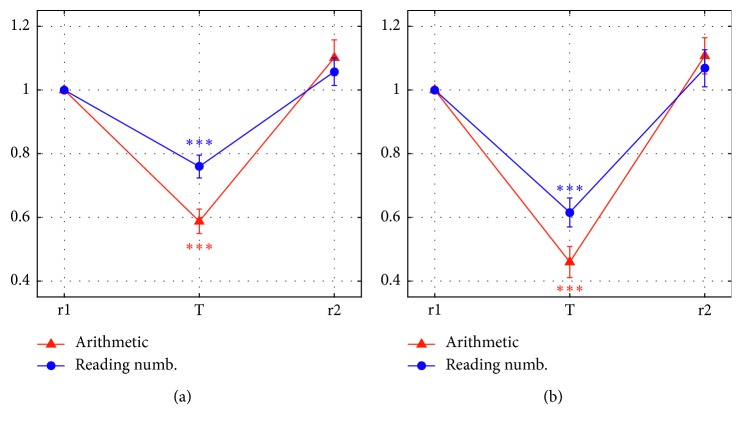
Normalized alpha power, averaged across participants (mean ± sem), over the two scalp regions (front-central-temporal FCT (a); parietal-occipital PO (b)) at each of the three phases (r1, T, and r2) of the arithmetic session and of the reading numbers session. Asterisks denote the results of multiple one-sample *t*-tests comparing the normalized alpha power in the phases T and r2 of each session with the reference value (1), separately within each region (significance cut-off = 0.05/4 = 0.0125). In both regions, significant deviation from the reference value was found during the task phases T (*p* < 0.0001 for both arithmetic and reading numbers) but not during the r2 phases (FCT: *p*=0.082 arithmetic; *p*=0.195 reading numbers; PO: *p*=0.07 arithmetic; *p*=0.25 reading numbers) (a) Normalized alpha power-FCT. (b) Normalized alpha power-PO.

The analysis at scalp-region level is presented in Figure 5 that depicts the normalized alpha power computed over the two scalp regions (FCT: Figure 5(a); PO: Figure 5(b)), in the three phases of the two experimental sessions (arithmetic/reading numbers). The values at phase T emphasize the stronger effect of the arithmetic task compared to the reading numbers task in reducing the alpha power within each region and the larger alpha power decrease in the PO region (Figure 5(b)) than that in the FCT region (Figure 5(a)) during each task. Multiple one-sample *t*-tests (Figure 5) confirmed that the normalized alpha power significantly deviated from the r1 reference value (1) during the task phase (both the arithmetic and reading numbers task), but not during the r2 phase, within each region. The 2 × 2 repeated measures ANOVA conducted on the normalized alpha power in T (factors: Task Type = arithmetic/reading and Region = FCT/PO) revealed that there was a main effect of Region (*F*(1,29) = 23.9, *p* < 0.0001), showing that the alpha power decreased more posteriorly than anteriorly during the tasks. Moreover, there was a main effect of Task Type (*F*(1,29) = 24.1, *p* < 0.0001) showing that the arithmetic task induced a larger alpha desynchronization than the reading numbers task.

### 3.2. Experiment 2: VR Immersion and Mental Task in VR Immersion

#### 3.2.1. Effect of the VR Immersion

This section presents the results obtained across the entire set of 41 participants, on phases r1, r1VR, and r2VR, showing the effects of the VR immersion in absence of any specific task. It is worth noticing that the displayed results concern the alpha power values over the two VR cabin sessions aggregated together (see Section 2.3.4 in Materials and Methods): indeed, the two virtual experiences turned out to be characterized by highly similar patterns of alpha powers (not shown results). This was an important preliminary outcome as it proved robustness of the adopted EEG measure and of its extraction procedure, attesting that similar VR configurations (hence similar visuospatial stimulations) induced similar alpha power changes.

**Figure 6:**
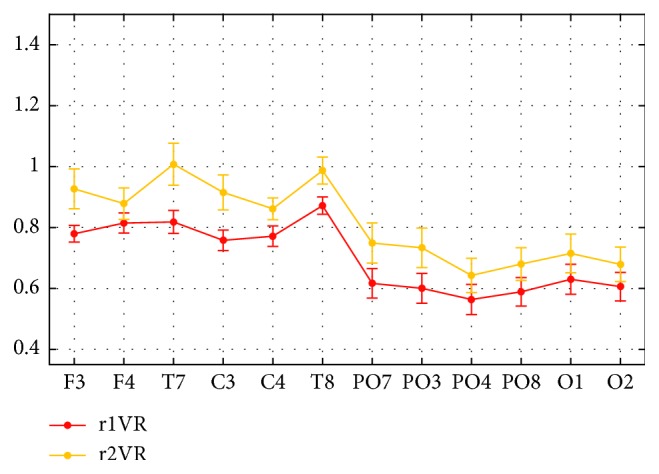
Normalized alpha power, averaged across participants (mean ± sem) and aggregated across the two sessions, at each single electrode in Experiment 2, during the two examined phases of purely VR immersion (r1VR and r2VR).

The analysis at single-channel level is shown in Figure 6; it plots the normalized alpha power at each electrode (mean ± sem across participants), during the phases of pure VR immersion (r1VR and r2VR). The following main observations can be drawn. First, the alpha power during the first visual exploration (r1VR, preinteraction) exhibited larger decrease than during the second visual exploration (r2VR, postinteraction) across all electrodes. Moreover, an abrupt decrease in the normalized alpha power occurred at the transition from the fronto-central-temporal to the parieto-occipital channels, both in r1VR and r2VR, while the electrodes within each set exhibited close values, similarly to what observed in Experiment 1 (Figure 4).

Motivated by the previous differences, an analysis at scalp-region level was performed in this case too.

**Figure 7:**
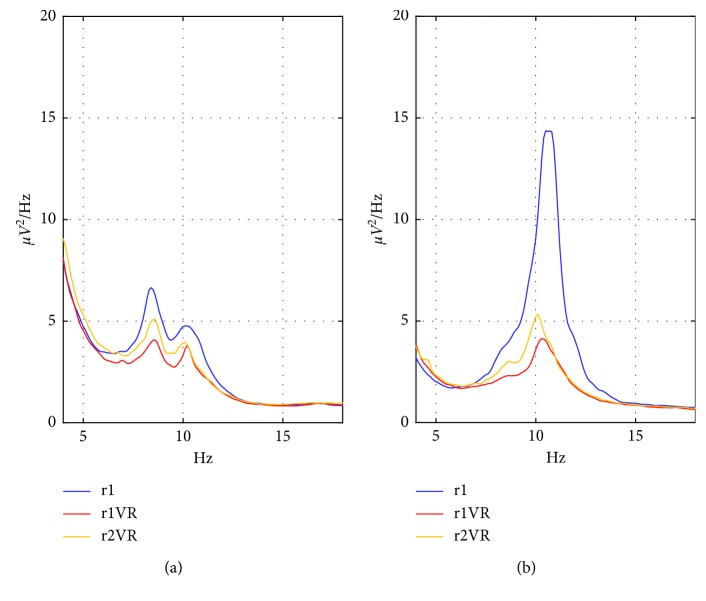
Power Spectrum Density (PSD) over each scalp region (front-central-temporal FCT (a); parietal-occipital PO (b)) computed separately for each phase r1, r1Vr, and r2VR, averaged across participants and across the two sessions (a) PSD-FCT. (b) PSD-PO.

Figure 7 shows the PSD over each scalp region (FCT region: Figure 7(a); PO region: Figure 7(b)) averaged across participants, and computed separately for each phase. The PO region (Figure 7(b)) was characterized by a huge peak in alpha band in the reference state r1 that dramatically decreased during r1VR and r2VR, while the FCT region (Figure 7(a)) presented a lower alpha peak and smaller modulation of its amplitude.

**Figure 8:**
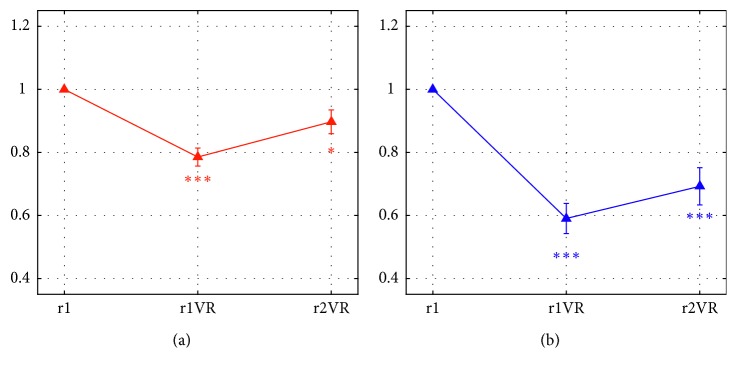
Normalized alpha power, averaged across participants (mean ± sem), over the two scalp regions (front-central-temporal FCT (a); parietal-occipital PO (b)) at each of the three phases (r1, r1VR, and r2VR) of the VR sessions. Asterisks denote the results of multiple one-sample *t*-tests comparing the normalized α powers in the phases r1VR and r2VR with the reference value (1), separately within each regions (significance cut-off = 0.05/2 = 0.025). In both regions, significant deviation from the reference value was found both in r1VR and r2VR (^*∗∗∗*^*p* < 0.0001; ^*∗*^*p*=0.0096). (a) Normalized alpha power-FCT. (b) Normalized alpha power-PO.

The obtained values of the normalized alpha power (mean ± sem across participants) in the three phases (r1, r1VR, and r2VR) are depicted in Figure 8, for each region separately (FCT: Figure 8(a); PO: Figure 8(b)). The VR immersion was characterized by a larger alpha power modulation over the PO region (Figure 8(b)) than the FCT region (Figure 8(a)). It is interesting to note that by comparing Figures 8 and 5, the alpha power desynchronization in VR immersion appeared to assume values similar to those observed in the reading numbers task rather than the arithmetic task, over both scalp regions. Multiple one-sample *t*-tests (Figure 8) confirmed a significant deviation of the normalized alpha power from the reference value (1) in both phases r1VR and r2VR, within each region. The 2 × 2 repeated measures ANOVA (factors: Phase = r1VR/r2VR and Region = FCT/PO) revealed that there was a main effect of Region (*F*(1,40) = 29.32, *p* < 0.0001) showing that alpha power decreased more posteriorly than anteriorly during the VR immersion. Moreover, there was a main effect of Phase (*F*(1,40) = 15.01, *p*=0.0004), indicating that alpha exhibited a larger desynchronization in the preinteraction static immersion (r1VR) than that in the postinteraction static immersion (r2VR).

**Figure 9:**
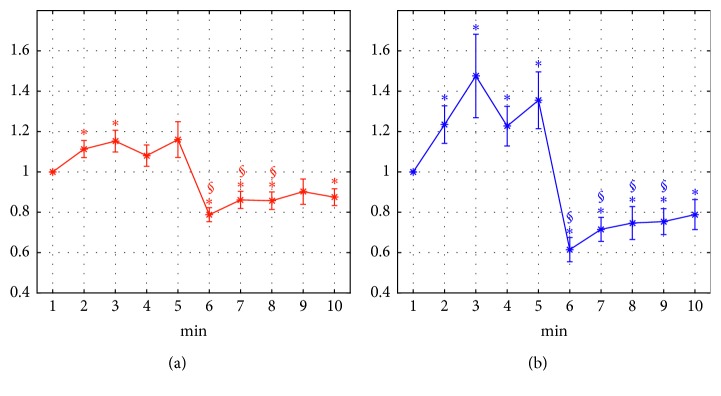
Temporal pattern, at 1-minute resolution, of the normalized alpha power (mean ± sem across participants) throughout the first ten minutes of the VR sessions, plotted separately for each scalp region (front-central-temporal FCT (a); parietal-occipital PO (b)). The examined ten minutes included the r1 phase from minute 1 to minute 5 and the r1VR phase from minute 6 to minute 10. For each region, the α power at each minute was normalized with respect to the value obtained at minute 1 (i.e., the first minute of phase r1). Symbols above each point denote the results of multiple one-sample *t*-tests comparing the normalized α power at each minute (from 2 to 10) with the reference value (1), separately within each region (significance cut-off = 0.05/9 = 0.0056). Symbols ∗ denote points that satisfied the uncorrected significance threshold (0.05), while symbols § denote points that survived the severe Bonferroni correction (0.05/9). Uncorrected *p* values at each point (subscript indicate the minute at which the *p* value refer to) are *p*_2_=0.015, *p*_3_=0.01, *p*_4_=0.159, *p*_5_=0.089, *p*_6_=1.5 · 10^−6^, *p*_7_=0.004, *p*_8_=0.003; *p*_9_=0.136, *p*_10_=0.006 for the FCT region; *p*_2_=0.021, *p*_3_=0.032, *p*_4_=0.036, *p*_5_=0.019, *p*_6_=4 · 10^−7^, *p*_7_=5 · 10^−5^, *p*_8_=0.004; *p*_9_=6 · 10^−4^, *p*_10_=0.011 for the PO region. (a) Normalized alpha power-FCT. (b) Normalized alpha power-PO.

Furthermore, we tested whether the alpha power index was able not only to capture differences among distinct 5-minute phases, but also to monitor trends and variations with a higher temporal resolution (1 minute), to promptly detect a change in the state of the participant at the transition from one phase to another.  Figure 9 plots the temporal pattern, at 1 min resolution, of the normalized alpha power (mean ± sem across participants) throughout the first ten minutes of the sessions (comprising phases r1 and r1VR), over each region (FCT: Figure 9(a); PO: Figure 9(b)). An interesting pattern emerged especially in the PO region (Figure 9(b)). In this region, alpha power exhibited an evident secondary increase after the first minute of the r1 phase. This pattern may reflect a progressive relaxation in the very first period of phase r1, when the participants were seated down and got used to the experimental setup. A large and abrupt alpha power decrease (evident also in the FCT region) occurred at minute 6, as soon as the participant got immersed in the VR environment, as an evident marker of visual stimulation and capture of external attention by the immersive sensory inputs. In the following minutes (minutes 7–10), the alpha power tended to moderately increase suggesting a gradual lessening of attention as the immersion in the static VR environment went on.  Figure 9 also displays the results of the multiple one-sample *t*-tests contrasting the normalized alpha power at each minute with the reference value (1), within each region. Almost all time points satisfied the uncorrected significance threshold (0.05, *∗*), except minutes 4, 5, and 9 in the FCT region. Interestingly, minutes from 6 to 8 (and even minute 9 in the PO region) survived the Bonferroni corrected threshold (0.05/9, §).

#### 3.2.2. Effect of an Internal Cognitive Task in VR Immersion

This section presents the results obtained across the subset of 24 participants, in the phases r1, r1VR, r2VR, and maVR of the second session, showing how the alpha power was modified when shifting from a condition of external attention to a condition requiring internal attention against the external appealing environment.

**Figure 10:**
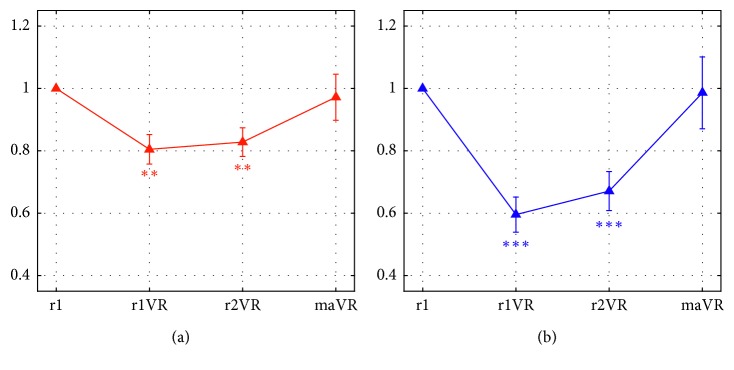
Normalized alpha power, averaged across the subset of 24 participants (mean ± sem), over the two scalp regions (front-central-temporal FCT (a); parietal-occipital PO (b)) in the four phases of the second session (r1, r1VR, r2VR, and maVR). Asterisks denote the results of multiple one-sample *t*-tests comparing the normalized α power in the phases r1VR, r2VR, and maVR with the reference value (1), separately within each region (significance threshold = 0.05/3 = 0.0167). In both regions, significant deviation from the reference value was found in r1VR and r2VR (FCT: ^*∗∗*^*p*=0.0004 in r1VR, ^*∗∗*^*p*=0.001 in r2VR; PO: ^*∗∗∗*^*p* < 0.0001 in r1VR and r2VR), but not in maVR (FCT: *p*=0.71; PO: *p*=0.9). (a) Normalized alpha power-FCT. (b) Normalized alpha power-PO.

Figure 10 displays the normalized alpha power (mean ± sem across the 24 participants) in the four phases, separately for the two regions (FCT: Figure 10(a); PO: Figure 10(b)). As well expected, the alpha power exhibited a decrease in both phases r1VR and r2VR, more evident in the PO region, similarly to the effects previously observed across all participants and sessions (Figure 8). Here, it is remarkable the dramatic increase in the alpha power induced by the execution of the mental arithmetic during the VR immersion. In particular, in this condition, the alpha power assumed values very close to the reference value, i.e., to the initial relaxation condition (r1). Indeed, the one-sample *t*-tests confirmed that the normalized alpha power during maVR did not deviate from the reference value (1), at variance with the phases r1VR and r2VR (Figure 10). The 3 × 2 repeated measure ANOVA (factors: Phase = r1VR/r2VR/maVR and Region = FCT/PO) disclosed that there was a significant Phase × Region interaction (*F*(2,46) = 10.77, *p*=0.0001) and a main effect of Phase (*F*(2,46) = 9.299, *p*=0.0004). Indeed, post hoc *t*-tests revealed that the alpha power was lower in the PO region than FCT region in both phases r1VR and r2VR (*p* < 0.0001 in both phases, corrected significance threshold = 0.05/3 = 0.0167), whereas no difference across the two regions emerged in the maVR phase (*p* > 0.999). Moreover, the alpha power in each phase, r1VR and r2VR, was lower than in maVR (*p*=0.0002 and *p*=0.0017, respectively; corrected significance threshold = 0.05/3 = 0.0167).

## 4. Discussion

The present results provide several interesting indications, which not only may contribute to our understanding of the role of alpha oscillations and of the mechanisms driving alpha increase/decrease, but can also have practical perspectives in future studies oriented to the noninvasive assessment of human/environment interaction via scalp EEG.

### 4.1. Electrodes Position

First, all electrodes in the scalp exhibited a significant ERD in the alpha band, both during the laboratory tasks (Experiment 1) and during the pure VR immersion. However, the level of ERD was significantly stronger in the parietal-occipital electrodes compared to the frontal-central ones. In particular, in these experiments, a drastic fall in alpha power was evident passing from the frontal-central to the parietal-occipital electrodes (Figures 4 and 6). This difference was even more evident using absolute values of power instead of normalized ones (Figure 3). This result agrees with results of several neurocognitive works. Indeed, recent EEG studies suggest that parietal and occipital regions are involved in visuospatial processing of stimuli [15, 42], spatial representations of numbers [51], and arithmetic problems [39, 43], at least when the latter involved external attention and visual processing too (such as the arithmetic task of the Experiment 1). It is probable, however, that other kinds of tasks (for instance those requiring motor actions or working memory) rely more on frontal-central regions [25, 52] and on other rhythms such as theta, beta, or gamma [53]. An interesting point is that the same electrodes (PO3 and PO4) were mainly sensitive both in the laboratory cognitive tasks and in the VR immersion (Figures 4 and 6). This provides the indication that, at least in this kind of problems, the number of electrodes can be significantly reduced without a significant loss in method sensitivity, thus further dropping the complexity of the experimental setup and improving its portability in real scenarios.

### 4.2. ERD and Attention

As we anticipated above, results of the present study confirm several data in the literature; however, they also introduce some interesting new elements. (1) First, we confirmed that attention to visual stimuli (either in the reading numbers task or in the Virtual Reality immersion) causes a significant ERD compared with a previous relaxation phase, especially accentuated in the parieto-occipital regions. Although various authors observed ERD in response to visual engagements [9–14], this is the first time that visual intake is not produced by specific stimuli, but via a full immersion in a motivating VR environment. This signifies that VR environments can represent a new important tool to study human internal vs. external attention in future work, more similar to conditions occurring in real life. (2) In Experiment 1, we differentiated the effect of a simpler visual task (pure reading numbers) and a more complex task (reading + arithmetic operation) which still involved external attention but a higher internal processing. In fact, from the previous analysis of the literature, it is still not clear whether and in which conditions an increase in the internal task produces ERS or ERD. Our results indicate that ERD was more accentuated during the demanding task (arithmetic operation), i.e., the arithmetic computation (although internal) further reduced alpha power, provided the task was driven by external visual inputs (attention to the digits). This result means that the alpha-band power can be finely modulated by the level of external attention and that external attention (not the task load) is the dominant factor in visual tasks. This result agrees with previous studies [3, 8]. Moreover, Schupp et al. [23] observed lower alpha for perceptual tasks as opposed to purely mental tasks. In agreement with our result, Benedek et al. [54] suggest that task processing under low internal processing demands (i.e., involving bottom-up processing) did not result in alpha synchronization but rather in strong desynchronization, especially in posterior brain regions, which could reflect stronger demands on the visual system. Only during more demanding tasks, involving top-down control and creativity, can ERS be verified. This result apparently disagrees with a result by Cooper et al. [21], who observed an increase in alpha power with the task demand not only during internal, but also during external attention tasks. We think that these differences may depend on the fact that, during Cooper et al. experiments, some items, given in sequence, should be maintained in memory for a certain period, whereas in our experiment, all numbers were simultaneously available and the external input stream dominated the process. In conclusion, our original result is that an internal arithmetic task can produce ERD, if dominated by external attention. (3) At odds with the previous experiment, in the VR experiment (maVR phase), we used an arithmetic task which was merely mental, while the strong visual intake (cabin immersion) had no relation with the task. At the same time, we did not use specific distractors, but the overall full immersion in the cabin environment had a distractor function. In this condition, we demonstrate that alpha power returned to approximately the same level (or in some participants, even to a higher level) as in the initial resting condition. This result agrees with previous studies, showing that alpha activity increases during a purely mental task not driven by sensory inputs [20, 22, 24]. A difference from previous studies, however, is that we started the mental task from a condition in which alpha power was already significantly reduced by attention to the cabin. We are not aware of any similar experiment performed before (i.e., a global environment distractor). It is interesting to observe that alpha power returned toward baseline (i.e., the resting state), suggesting that the participant was trying to completely neglect the VR environment, i.e., to reach a complete isolation state. This result suggests that the alpha power increase has the most important function to isolate a subject from the external world.

### 4.3. Artefact Removal

EEG signals are commonly affected by artefacts. Hence, artefact removal is an important aspect of any EEG processing method. Today, ICA is probably the most employed method for removal of artefactual activity from EEG cerebral signals [44], being highly effective in separating several stereotyped nonbrain artefacts (eye blinks, eye movement potentials, EMG, and ECG) from the rest of EEG signals, given that they represent independent physical processes. For this reason, we used this classical and consolidated technique to effectively remove artefacts from the EEG recording acquired in the controlled laboratory setting (Experiment 1) via the wired, laboratory-grade device. However, accurate EEG artefact removal in environments outside controlled laboratory settings, in real or realistic scenarios, and/or in online applications, is still a critical open issue. In these less-controlled conditions, indeed, several nonstereotyped and transient artefacts may corrupt the EEG signals, and ICA may result ineffective in separating them if not sufficient stationary time points are provided. Indeed, we encountered this problem in our recordings obtained in the VR environment with the wireless, consumer-grade EEG device: a large number of artefactual elements (including several nonstereotyped activities) were mixed over most or all ICs, making it impossible to separate the useful signal from the spurious noise via a simple IC selection. This problem is further aggravated (as in our recordings) when a limited number of EEG channels is acquired, as the number of estimated ICs, in the basic ICA model, is constrained to be equal to the channel number (thus imposing a superior limit to the number of independent signals that can compose the mixed EEG for their efficient separation). On the other hand, a limited number of electrodes is a desirable feature in real applications reducing the time of preparation and cost. Due to the ineffectiveness of ICA, in case of the VR recordings, we simply eliminated the EEG portions affected by too much noise from the signal processing procedure: portions with too much noise were not examined and did not contribute to the final analysis. Results, however, were still quite robust and reliable as shown in Figures 6–9. Moreover, the robustness of our procedure for EEG processing in VR recordings was further supported by our preliminary analysis performed on the results obtained separately in the B1 and B2 virtual configurations; this analysis (not shown results) verified that the two virtual configurations, pretty similar and thus eliciting similar visuospatial sensory stimulation, induced overlapped patterns of EEG alpha powers. However, our study confirms that EEG artefactual removal is still a crucial problem in real-world or realistic applications. This problem is currently faced by the scientific community and new methods, also more online-capable than ICA, for removal of transient, and nonstereotyped artefacts have been recently suggested [44, 55]. An important development of the present study will concern testing alternative and more recent methods other than ICA for artefact correction of the VR recordings.

### 4.4. Temporal Aspects

During the VR immersion, the participant experienced a phase in which he/she was fully immersed in the cabin environment (r1VR), simply sitting down as a passenger during a travel, followed by a second phase in which he/she moved along the environment interacting with the objects (intVR). Then, a third phase followed, in which he/she seated again in a relaxed state fully immersed in the visual and acoustic cabin details (r2VR). We did not use the EEG registered during the interaction with the cabin, since the rapid body and head movements produced too much artefact noise on the electrode signals. However, as anticipated above, it is interesting to observe that, in the third phase of the measurement (r2VR), when the participant sat again after the active interaction, alpha ERD was less evident compared with the first phase, and this difference was statistically significant (see Results in Figure 8 and corresponding ANOVA). This may indicate that the attention-grabbing effect that the VR scenario caused on the participant partially declined as the participant became more used to the environment.

Finally, we tested whether the method can detect mental state changes in a rapid temporal basis. To this aim, we computed the alpha power spectral density in one-minute intervals. Results show that rapid ERD can be detected fairly well and that the temporal variations detected have a straightforward interpretation. During the second minute of the initial relaxation, alpha power exhibited an evident increase, denoting that the participant was becoming more used to the experimental setup (and so was more relaxed). A rapid decrease in alpha-power was immediately observable after 5 minutes (Figure 9), i.e., in the first minute (minute 6) when the participant experienced his/her first immersion in the virtual reality. During the subsequent four minutes of immersion, the alpha power remained low, but showed a moderate temporal increase, reflecting a modest progressive reduction in the level of attention, that is a kind of settling. It is worth noticing that the variations captured by the alpha power index at 1 min resolution were statistically significant and especially large and consistent across the participants in the very first minutes following the VR immersion (Figure 9).

### 4.5. Analysis in the VR and Perspective Implications

An important point of strength of the present study is the specific investigation in the VR immersion. This analysis has evidenced that even a resting immersion in a static VR scenario had profound effect on EEG alpha power, as the rich sensory stimulation probably exerts a strong attention-grabbing influence, and this effect was quantitatively comparable to a high-level cognitive processing such as reading numbers. The assessment of EEG consequences of pure VR immersion is relevant to enhance interpretation of brain rhythms modifications when a subject is immersed in a complex realistic scenario. This may have perspective implications considering the emerging use of VR associated with EEG-based measures in several applications. In particular, the use of VR technology together with objective physiological measures (other than subjective evaluation) is rapidly increasing as a valuable tool to inform design decisions in the early phases of artificial environment projects ([56–59]) and/or to study human/environment interaction [60, 61]. Moreover and in a different context, there is a growing number of studies investing the use and effectiveness of VR-based therapy for psychiatric disorders [60, 62]. Of course, in all these applications, understanding how the simple immersion in a VR scenario (or a task performed in the VR) can modify the subject's physiological parameters, and in particular EEG parameters, is strictly necessary for a correct interpretation of the behavioural data and of the psychophysiological effects.

### 4.6. Limitations and Future Improvements

While the present study may provide interesting cues on the role of alpha oscillations and its relations with internal/external attentional components and task load, the objective of the present work was not to investigate the neural bases of alpha rhythm changes. In order to investigate the underlying neural mechanisms, more sophisticated methods should be implemented, with the use of high-density EEG recording, source reconstruction in the cortex, and estimation of connectivity changes between regions of interest. This may be the subject of subsequent studies.

Furthermore, in this work, we showed that traditional methods (like the Welch periodogram, computed on a shifting temporal window) can acceptably detect temporal changes of a nonstationary signal. However, time changes can be even better detected using more sophisticated processing methods, such as wavelets (as used, for instance, in [63] to build sensitive indicators of mental workload). This may be implemented and tested in future developments. Indeed, efficient algorithms for wavelet computation do exist, which are even compatible with real-time application.

## 5. Conclusions

In conclusion, emphasis in the present work is on the possibility to detect changes in attention during human/environment interaction, with the use of a simple unexpansive EEG technique, applicable in an artificial setting and prospectively in real time. The results emphasize that alpha power decreases during tasks which necessitate attention to the external environment, even in conditions when the task requires an increasing mental effort. Conversely, alpha power increases to levels similar to a relaxation state, when a task requires isolation from the external world. These results elucidate some aspects still insufficiently clear in the recent literature, suggesting that one of the main roles for the alpha rhythm is isolation from the external environment and attentional shift toward internal aspects.

A peculiarity of our study is the use of a sophisticate virtual reality environment to mimic the interaction of individuals with an artificial ad hoc designed scenario. In perspective, this may be used in the design of artificial systems, or in neuroengineering applications. Indeed, the possibility to monitor attentional changes from low-resolution EEG is of the greatest value to realize easy-to-use, comfortable, and cheaper systems in practical applications such as neurofeedback, brain computer interfaces, neuroergonomics, and neuromarketing [64–66].

## Data Availability

The data used to support the findings of this study are available (in anonymized form) upon request submitted to Elisa Magosso (elisa.magosso@unibo.it) and Francesca De Crescenzio (francesca.decrescenzio@unibo.it).
